# New Levan-Based Chiral Stationary Phases: Synthesis and Comparative HPLC Enantioseparation of (±)-*trans*-β-Lactam Ureas in the Polar Organic Mode

**DOI:** 10.3390/molecules29102213

**Published:** 2024-05-09

**Authors:** Darko Kontrec, Mladenka Jurin, Andreja Jakas, Marin Roje

**Affiliations:** Laboratory for Chiral Technologies, Division of Organic Chemistry and Biochemistry, Ruder Bošković Institute, Bijenička Cesta 54, 10 000 Zagreb, Croatia; darko.kontrec@irb.hr (D.K.); andreja.jakas@irb.hr (A.J.)

**Keywords:** levan, *trans*-β-lactam ureas, chiral separation, enantioselective HPLC, polar organic mode, polysaccharide-type chiral stationary phases, CSPs, new chiral material

## Abstract

In this paper, the preparation of three new polysaccharide-type chiral stationary phases (CSPs) based on levan carbamates (3,5-dimethylphenyl, 4-methylphenyl, and 1-naphthyl) is described. The enantioseparation of (±)-*trans*-β-lactam ureas **1a**–**h** was investigated by high-performance liquid chromatography (HPLC) on six different chiral columns (Chiralpak AD-3, Chiralcel OD-3, Chirallica PST-7, Chirallica PST-8, Chirallica PST-9, and Chirallica PST-10) in the polar organic mode, using pure methanol (MeOH), ethanol (EtOH), and acetonitrile (ACN). Apart from the Chirallica PST-9 column (based on levan *tris*(1-naphthylcarbamate), the columns exhibited a satisfactory chiral recognition ability for the tested *trans*-β-lactam ureas **1a**–**h**.

## 1. Introduction

Chiral separation has attracted much attention in the last thirty years due to the important physiological and biological activities of enantiomers [1,2,3]. Among the various separation techniques, such as gas chromatography (GC) [4,5], high-performance liquid chromatography (HPLC) [6,7], supercritical fluid chromatography (SFC) [8,9,10], capillary electrophoresis (CE) [11,12,13], capillary electrochromatography (CEC) [14,15,16], and nano liquid chromatography (nano-LC) [17], the HPLC based on the application of chiral stationary phases (CSPs) is a powerful tool for the analysis of enantiomers, as well as for obtaining enantiomerically pure compounds on a preparative scale [18].

Many CSPs have been developed, including brush-type, ligand-exchange, cyclodextrin, macrocyclic antibiotics, protein, and polysaccharide types [19,20,21,22,23]. Among these CSPs, polysaccharide-type CSPs, especially amylose and cellulose-derivative CSPs, have been shown to be one of the most useful CSPs due to their excellent performance in chiral separation [24]. In addition to amylose and cellulose, derivatives of other polysaccharides, such as chitin [25], chitosan, galactosamine, curdlan, dextran, xylan, and inulin, can also be used as chiral stationary phases, but their chiral recognition abilities as CSPs for HPLC enantioseparation depend on the nature of the monosaccharide units, linkage position, and linkage type [26].

**Figure 1:**
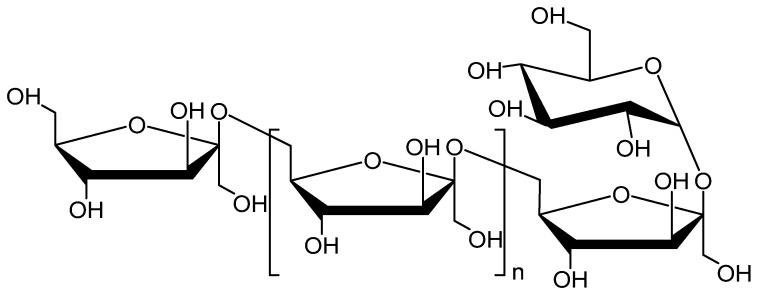
Chemical structure of levan isolated from Timothy grass (*Phleum pratence*).

Levan is a fructan polymer consisting of β-2,6 linked fructofuranose residues in a linear chain with intermittent β-2,1-linked side chains [27,28,29]. The molecular weight and the degree of branching depend on the source of the levan. Levans with low molecular weight and minimal branching can be found in grasses [30]. In contrast, the levan produced by bacteria is characterized by a high molecular weight, which can exceed 10^7^ [31], and it contains side chains on up to 30% of the main chain residues [32]. The conformation of levan has been far less studied than other common polysaccharides, such as amylose and cellulose. The conformational properties of the levan have been investigated by computer modeling, indicating that the helices of levan are left-handed [33]. These structural features make levan a possible candidate for the role of an effective chiral selector in CSPs. The polysaccharide levan (Figure 1) used in this study is derived from the ubiquitous Timothy grass, *Phleum pratence*, and is an easily accessible and relatively inexpensive chiral material of natural origin.

Polysaccharide carbamate derivatives were originally proposed as CSPs for normal-phase HPLC [24,34,35,36,37]. It was later shown that these materials can also be used effectively in reversed-phase chromatography [19,34,38]. In addition to these two modes, these materials can also be used in polar organic mode, which has become increasingly popular for various CSPs in recent years [34,38,39,40]. The polar organic mode is particularly attractive for the analytical and preparative separation of enantiomers, as it offers several advantages, such as a shorter analysis time, high efficiency, better signal/noise ratio, usually higher solubility of the analytes in the mobile phase, etc. [39,40,41,42]. In the polar organic mode, only polar organic solvents (except water), usually alcohol (methanol, ethanol, and 2-propanol), acetonitrile, or their combinations are used as the mobile phase [39,40,41].

β-lactams, a common name for azetidin-2-one, are organic four-membered cyclic amides [43] and are widely used in medicinal and organic chemistry [44]. β-lactam-containing compounds exhibit a broad spectrum of pharmacological and biological activities, such as antibacterial [43], anticancer, antiviral [45], antitubercular, anti-HIV, anti-inflammatory, and other biological activities [46,47]. In addition, these compounds act as potent inhibitors of serine protease, human leukocyte elastase, and human cytomegalovirus protease enzymes [46]. Optically active β-lactams are versatile building blocks that have found widespread use in organic synthesis [48], such as the preparation of β-amino acids, peptides, peptidomimetics, taxoids, alkaloids, and various heterocyclic molecules [44].

Chiral HPLC based on the application of chiral stationary phases (CSPs) is an effective tool for the separation of β-lactam enantiomers on the brush-type [49,50], polysaccharide-based [51,52,53,54,55,56], cyclodextrin-based [57,58,59], or macrocyclic glycopeptide-based CSPs [54,58].

Recently, we reported the enantioseparation of β-lactam ureas on immobilized amylose and cellulose phenylcarbamate-based CSPs by HPLC in the normal phase, polar organic phase, and non-standard phase modes [56]. We also reported enantioseparation on coated and/or immobilized amylose- and cellulose-based CSPs by SFC with the typical mobile phase CO_2_/alcohol [55,56] and with the atypical mobile phase consisting of CO_2_/dimethyl carbonate/alcohol mixtures [56].

**Figure 2:**
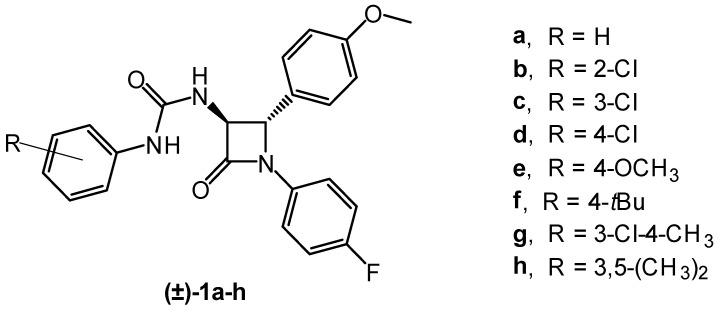
Chemical structures of (±)-*trans*-β-lactam ureas **1a**–**h**.

The present study describes the preparation of three novel polysaccharide-type chiral stationary phases based on low-molecular-weight levan derivatives coated on a wide-pored silica gel. Their ability to enact the enantioseparation of (±)-*trans*-β-lactam ureas **1a**–**h** (Figure 2) was evaluated and compared with the two most commonly used commercially available polysaccharide-type CSPs, Chiralpak AD and Chiralcel OD, and a less studied cellulose derivative, namely cellulose *tris*(4-methylphenylcarbamate) (Chirallica PST-10). 

## 2. Results and Discussion

### 2.1. Characterization of Chiral Selectors and Chiral Stationary Phases

**Figure 3:**
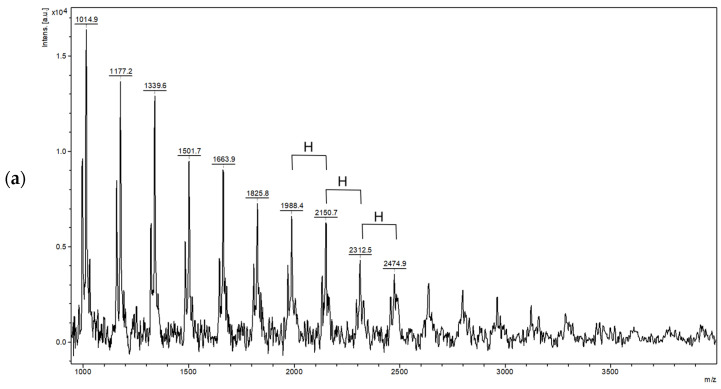

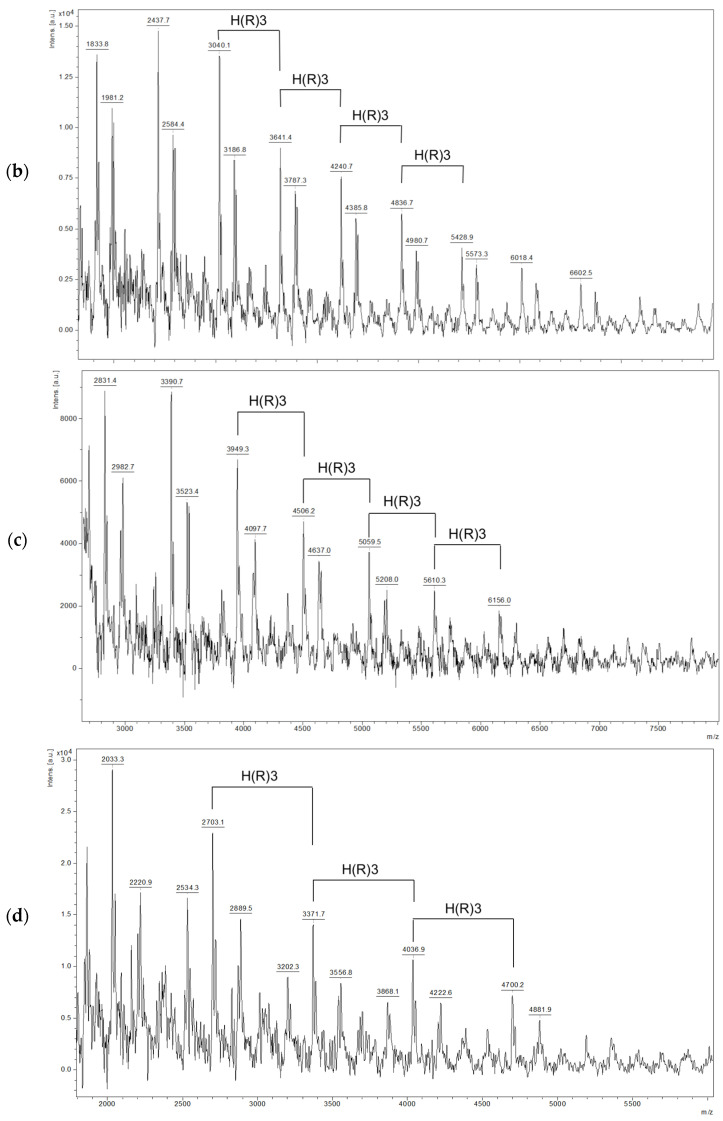
MADLI-TOF MS spectra of levan (**a**), levan *tris*(3,5-dimethylphenylcarbamate) (**b**), levan *tris*(4-methylphenylcarbamate) (**c**), and levan *tris*(1-naphthylcarbamate) (**d**). H = hexoses unit; R = 3,5-dimethylphenylcarbamate (**b**), R = 4-methylphenylcarbamate (**c**), and R = 1-naphthylcarbamate (**d**).

Figure 3 shows the degree of substitution of the hexoses of the polysaccharide levan. Figure 3a shows the MALDI-TOF MS spectra of levan and the difference in masses of 162 Da for each hexose sugar unit (H). Figure 3b shows the MALDI-TOF MS spectra of levan *tris*(3,5-dimethylphenylcarbamate) and the difference in mass of 603 Da for the persubstituted sugar unit (H(R)3), for levan *tris*(4-methylphenyl-carbamate ~561 (Figure 3c), and for levan *tris*(1-naphthylcarbamate) ~669 Da (Figure 3d).

**Figure 4:**
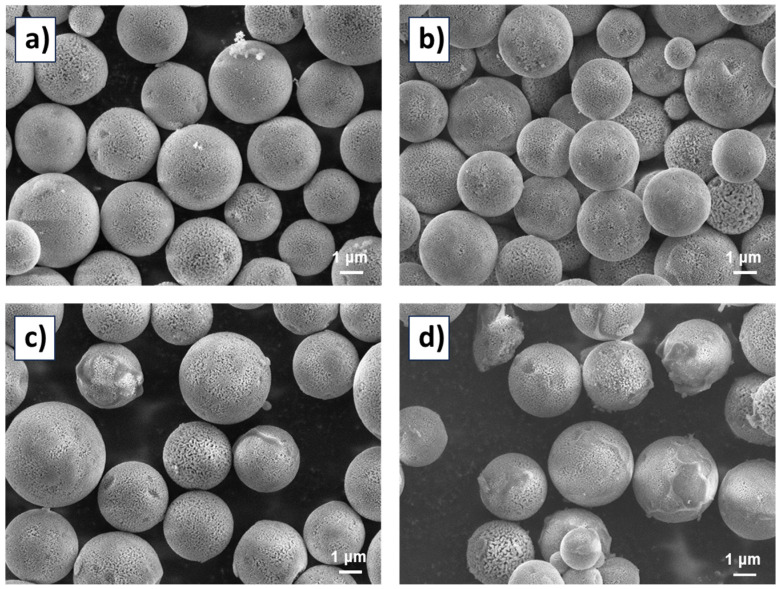
SEM images of the studied silica particles: (**a**) original silica particles, (**b**) CSP based on levan *tris*(3,5-dimethylphenylcarbamate), (**c**) CSP based on levan *tris*(4-methylphenylcarbamate), and (**d**) CSP based on levan *tris*(1-naphthylcarbamate).

The morphologies of the silica gel Daisogel SP-1000-5-C1 and CSPs based on levan carbamate derivatives were investigated by scanning electron microscopy (SEM). The particles in all samples present with a spherical morphology; their diameter ranges from 1 to 10 μm. The surface of the particles is not smooth; voids that are present on the outer surface of the particles can be observed in all systems. Some of the particles in Figure 4c,d are coated with a thin layer.

### 2.2. Enantioseparation of trans-β-Lactam Ureas ***1a**–**h***

The six chiral selectors used in this work differ from each other in terms of the polysaccharide backbone (amylose, cellulose, or levan) and/or the type of the substituent (3,5-dimethylphenyl, 4-methylphenyl, and 1-naphthyl). The columns Chiralpak AD-3, Chiralcel OD-3, and Chirallica PST-7 differ only in the polysaccharide backbone, while Chiralcel OD-3 and Chirallica PST-10, as well as Chirallica PST-7, Chirallica PST-8, and Chirallica PST-9, differ only in regard to the type of substituents (Figure 3). These differences in the molecular structure of the monomers have a major influence on the three-dimensional structures of the chiral polymers. It is assumed that the differences in chiral recognition mechanisms in polysaccharide-type selectors are strongly related to the differences in the molecular environment of the chiral cavities in these selectors. 

The chiral selectors based on levan and cellulose were prepared using a standard chemical derivatization protocol that is also used for the preparation of chiral selectors based on amylose or cellulose carbamates [60]. Under these conditions, the persubstituted levan or cellulose derivatives are the expected products, as confirmed by MALDI-TOF mass spectrometry in the case of levan-based chiral selectors (Figure 3).

The compounds **1a**–**h** investigated in this study (Figure 2) have the same β-lactam ring with two chiral carbon atoms (C-3 and C-4 atoms of the β-lactam ring). These compounds differ only in the type or position of the substituents on the phenyl ring attached to the nitrogen atom of the ureido moiety. All of these different structural features can influence the molecular properties (e.g., size and polarity of the solute) and thus influence the interactions between the selector and the analyte. 

**Figure 5:**
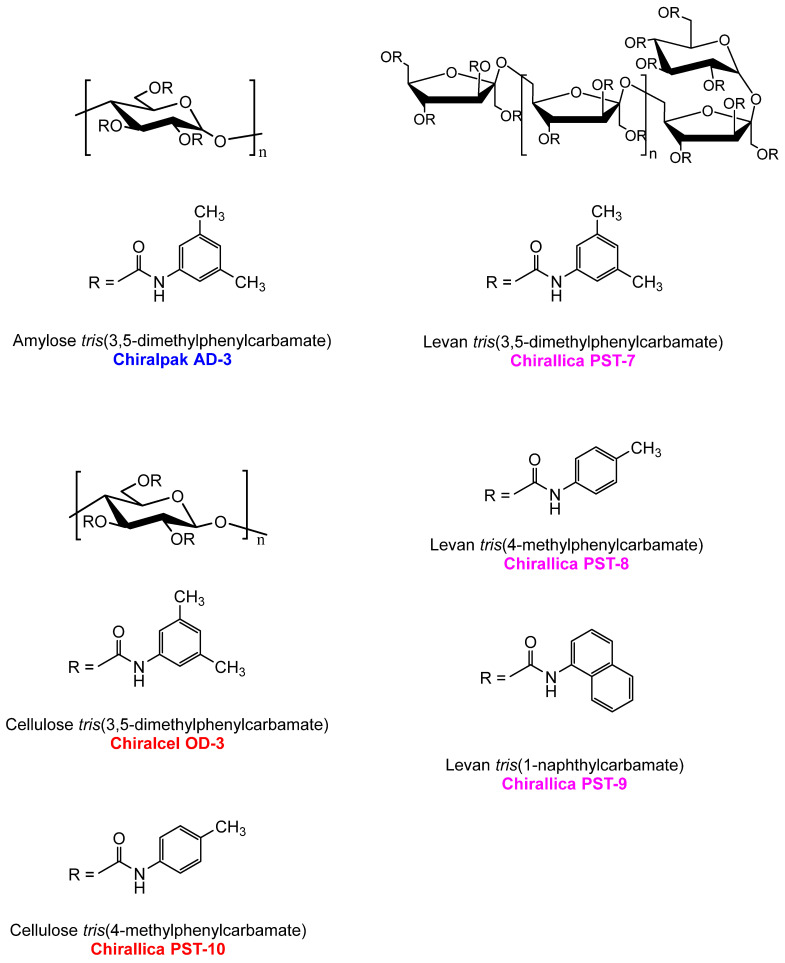
Chemical structures of carbamate derivatives of amylose, cellulose, and levan.

The chiral recognition mechanism of polysaccharide-based CSPs is much more complex, as their chiral recognition usually depends on their supramolecular structure and the steric fit of the analyte within the chiral cavity [34,61]. The carbonyl group (C=O), the –OCH_3_ and –NH groups, and the phenyl moiety in the structure of chiral β-lactam compounds can form a hydrogen bond, a dipole–dipole bond, π-π interactions, and hydrophobic interactions with the C=O, the –NH group, and the aromatic ring of CSPs. The π-electron density, steric hindrances, different conformations, and spatial structures of the polysaccharide-glucose or fructose moiety on the CSPs influence the interaction between the CSPs and the tested chiral β-lactam ureas. The different types and intensities of the interactions between the β-lactam compounds and the CSPs led to different chiral recognition phenomena. The chemical structures of the six chiral selectors are shown in Figure 5.

Chromatographic parameters such as the retention factor of the first eluting enantiomer (*k*_1_), separation factor (*α*), and resolution (*R*_s_) are summarized in Table 1. 

The Chiralcel OD-3 column showed a stronger ability for enantioseparation compared to the Chiralpak AD-3 and Chirallica PST-7 columns for *trans*-β-lactam ureas **1a**–**h**. These three columns contain the same chiral selector (3,5-dimethylphenylcarbamate moiety) but differ in the polysaccharide backbone, namely cellulose, amylose, and levan, respectively. When using the Chirallica PST-7 column, the retention factors of the first eluted enantiomers (*k*_1_) were lower than they were for the amylose and cellulose equivalents. However, the stronger interaction did not always result in higher values of the resolution (*R*_s_) and enantioselectivity factor (*α*). In the case of Chirallica PST-7, the enantioselectivity was higher, although the retention factors were the lowest. The Chiralcel OD-3 showed significantly higher resolution values for all tested ureas compared to the Chiralpak AD-3 and Chirallica PST-7 columns. From these results, it appears that the chiral recognition mechanism depends on the higher-order structure of the polysaccharide used. The same applies to the Chirallica PST-10 column and the Chirallica PST-7 column, which contain the same 4-methylphenylcarbamate substituent bound to cellulose and levan, respectively. On the Chirallica PST-10 cellulose column, the retention time of the first eluted enantiomers and the resolution values were greater than those of the levan analog, the Chirallica PST-7 column. The performance of Chirallica PST-7 (levan *tris*(3,5-dimethylphenylcarbamate)) is also satisfactory, as it partially or baseline separates all tested compounds **1a**–**h**. Its enantioselectivity and resolution values were higher than those of the other two levan CSPs. The Chirallica PST-8 column was also effective, being able to partially separate seven β-lactam ureas (**1a**–**f** and **1h**) and one to baseline (compound **1g**). The third levan column Chirallica PST-9 was not satisfactory, as no chiral separation was observed for half of the compounds tested. The chiral recognition ability towards the tested compounds **1a**–**h** depended markedly on the substituents on levan-based CSPs.

The effects of the nature and position of the substituent on the aromatic ring were investigated in the polar organic mode on all polysaccharide columns used. The electron-donating methoxy or *tert*-butyl substitution on the benzyl ring (**1a** vs. **1e**, and **1f**) led to significant variations in the chromatographic values. In the case of the methoxy group in *para*-position on the phenyl ring (**1e**), the *k*_1_ and *R*_s_ values were higher compared to the unsubstituted phenyl ring **1a** (except for the Chirallica PST-7 column). The *tert*-butyl substitution of the aromatic ring in *para* position led to higher *k*_1_, *α*, and *R*_s_ values on the cellulose column Chiralcel OD-3 and on all three levan columns used.

In contrast, substitution with an electron-withdrawing chlorine atom (**1a** vs. **1d**) also led to variations in the chromatographic values; however, these variations depend on the applied conditions (i.e., on the CSP).

It was found that the position of the chlorine atom in **1b** compared to **1c**, and **1d** significantly affects chiral recognition. The chlorine atom placed in the *ortho* position led to shorter retentions and, in most cases, to reduced enantiorecognition on cellulose Chiralcel OD-3, as well as Chirallica PST-10, and levan Chirallica PST-7 CSPs. The halogen substitution of the aromatic ring in the *ortho* position can sterically hinder effective chiral recognition in the case of the investigated β-lactam ureas on the cellulose and levan-based CSPs. Meanwhile, the chlorine atom placed in the *ortho* position **1b** led to a higher resolution on the amylose-based CSP. It appears that the compound with the chlorine atom in the *ortho* position of the phenyl ring fits better into the chiral cavities of amylose than the compound with the chlorine atom in the *meta* or *para* position of the phenyl ring (**1c** and **1d**).

As can be seen from these results (Table 1), most of the columns were suitable for the enantioseparation of β-lactam ureas **1a**–**h** using pure methanol as mobile phase.

In addition, the enantioseparations of these compounds were exanimated with less polar alcohol ethanol and aprotic acetonitrile. With the Chirallica PST-9 based on levan *tris*(1-naphthylcarbamate), no chiral separation was observed with ethanol and acetonitrile as the mobile phase.

On the Chiralpak AD-3, from eight chiral analytes examined with ethanol as the mobile phase, enantioselectivity was observed for six (Table 1). However, the baseline enantioseparation was observed for only five compounds **1a**–**f**. For example, **1f** was baseline resolved on the Chiralpak AD-3 column using ethanol with *R*_s_ = 6.06, but no enantiorecognition was observed with methanol. In the case of **1g**, partial separation with methanol was observed, but no separation with ethanol. The enantiomers of **1i** were not separated with either methanol or ethanol. The Chiralpak AD-3 column showed a slightly higher chiral recognition ability in acetonitrile compared to alcoholic mobile phases. Thus, of the eight chiral analytes studied with acetonitrile as the mobile phase, enantioselectivity was observed for all eight compounds (Table 1). However, a baseline enantioseparation was only observed for a few analytes. In contrast, of the eight chiral β-lactam ureas analyzed, the enantiomers of six compounds were separated in methanol and ethanol on this amylose-based column.

The columns Chiralcel OD-3 and Chirallica PST-7, which contain the same phenylcarbamate moiety as the column Chiralpak AD-3 column but are bound to the cellulose or levan backbone instead of amylose showed a rather high enantiomer separation ability compared to their amylose analog with methanol and ethanol as the mobile phase (Table 1). On the Chirallica PST-7 column, baseline separation was observed for all tested compounds **1a**–**h** in ethanol, while on the Chiralcel OD-3 column, seven compounds showed baseline separation. On the Chiralcel OD-3 with acetonitrile, baseline separation was observed for only four *trans*-β-lactam ureas, **1c**, **1d**, **1f**, and **1g**, while the partial resolution was observed for other four tested ureas, **1a**, **1b**, **1e**, and **1h**. Acetonitrile, in general, was less suitable compared to methanol and ethanol as the mobile phase with Chirallica PST-7 column. The baseline enantioseparation was only observed for five compounds, **1a** and **1c**–**f**.

All tested compounds, **1a**–**h**, were baseline separated on the Chirallica PST-10 column, based on cellulose *tris*(4-methylphenylcarbamate), with methanol and ethanol as the mobile phase. This column showed the highest resolution values with methanol and ethanol as the mobile phase compared to the other columns tested (except **1b** in ethanol). The enantioseparation of the synthesized *trans*-β-lactam ureas **1a**–**h** on the Chirallica PST-10 column was not investigated with acetonitrile due to the high column back pressure (above 40 MPa at 0.3 mL min^−1^) while using this mobile phase.

The comparison of the alcoholic eluents on the Chirallica PST-8 showed that the highest separation and resolution values were obtained with the less polar alcohol ethanol, while methanol appears to be the least favorable for the enantioseparation of these types of compounds. High enantioselectivities and resolution factors for five *trans*-β-lactam ureas, **1a**–**e**, in acetonitrile were observed on the Chirallica PST-8 compared to methanol. For example, the enantiomers of **1f**–**h** were separated in methanol and ethanol, but no enantioseparation was observed in acetonitrile.

At this stage, it was difficult to find clear dependencies between the structure and retention or separation factor. On the same column, some of the analytes were retained longer in methanol and others in ethanol or in acetonitrile as a mobile phase. The same was true also for the separation factor. This finding indicates that the intermolecular forces involved in sample retention and enantioseparation are multivariate. Thus, the chiral recognition mechanism depends not only on the mobile and chiral stationary phase, but also on the nature of analyzed compound.

**Figure 6:**
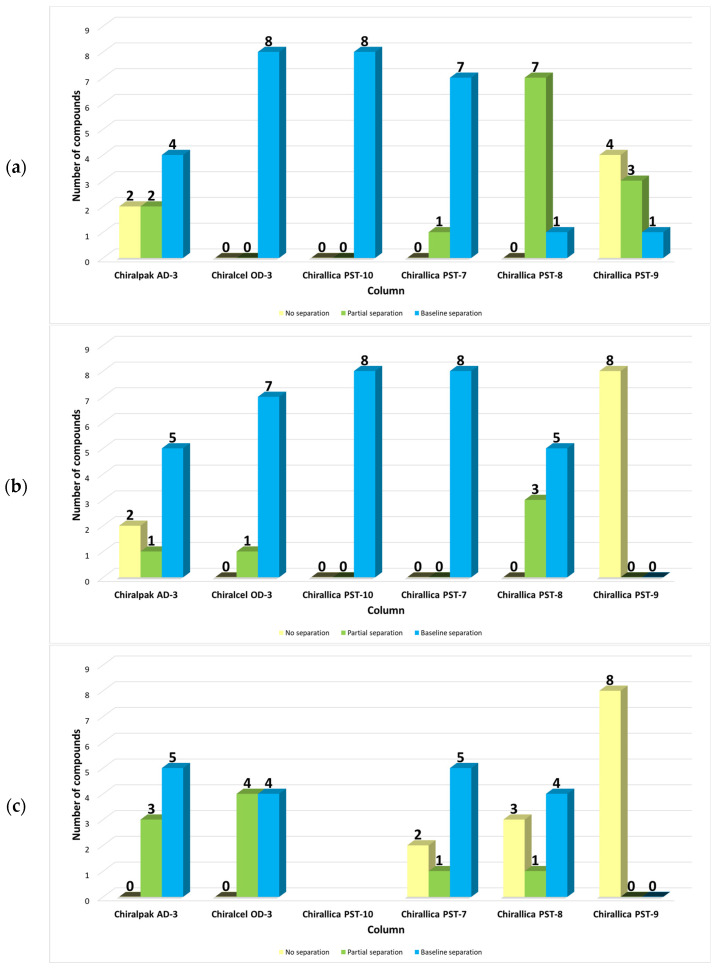
Efficiency of the six polysaccharide-based chiral columns in the separation of the set of eight racemic *trans*-β-lactam ureas **1a**–**h** in the polar organic mode, using 100% MeOH (**a**), 100% EtOH (**b**), and 100% ACN (**c**). Flow rate: 1 mL min^−1^.

Figure 6 summarizes the results of the enantioseparation screening of the *trans*-β-lactam ureas **1a**–**h**, using methanol, ethanol, and acetonitrile as the polar organic phase. Shown is the number of enantiomer separations for each column, with three bars indicating the number of non-separations, partial separations, and baseline separations. When the resolution is between 0 and 1.50, partial separation is achieved, indicating some degree of enantiorecognition of the chromatographic system (which is a combination of a CSP and a mobile phase) toward the compound [62]. For racemic compounds where *R*_s_ is ≥1.50, baseline resolution is achieved. The two columns that performed best in methanol were the Chiralcel OD-3 and the Chirallica PST-10, both of which were able to separate eight chiral β-lactam ureas or 100% of the test set shown in Table 1. The two levan columns, Chirallica PST-7 and Chirallica PST-8, also separated all ureas tested, but with a lower number of baseline separations. The Chirallica PST-9 column performed worse than the other two levan columns; baseline separation was only observed for four racemates (50%) with pure MeOH as the mobile phase. The Chiralpak AD-3 column, which contains the same phenylcarbamate moiety as the Chiralcel OD-3 column but is bound to the amylose backbone instead of cellulose, showed a lower enantiomer separation ability (75% in methanol and ethanol) compared to its cellulose analog (100% with both alcohol). In ethanol, the Chirallica PST-7 and Chirallica PST-10 columns performed best, as the baseline separations were observed for all eight compounds tested (100%). The Chirallica PST-8 separated all *trans*-β-lactam ureas tested (100%), but baseline separation was observed only for five compounds. No chiral separation was observed on the Chirallica PST-9 column when ethanol and acetonitrile (0%) were used as mobile phases. The two columns that performed the best in acetonitrile were the Chiralpak AD-3 and the Chiralcel OD-3, both of which separated all eight *trans*-β-lactam ureas (100%). The Chiralpak AD-3 had five baseline separations, and the Chiralcel OD-3 had four baseline separations. The Chirallica PST-7 resolved the enantiomers of six compounds, with five of them being baseline separated. The Chirallica PST-8 separated the enantiomers of five compounds, four of which had a baseline separation.

The Chiralcel OD-3 and Chirallica PST-10 columns, both cellulose-based stationary phases, and the Chirallica PST-7, a levan-based stationary phase, were found to be the best HPLC stationary phases for the enantioseparation of these eight *trans*-β-lactam ureas, **1a**–**h**, when methanol and ethanol were used as mobile phases. These three chiral stationary phases separated the largest number of compounds of all HPLC columns tested and exhibited the most baseline separations in methanol and ethanol. The Chiralpak AD-3 and its cellulosic analog, Chiralcel OD-3, appear to be the most suitable for the separation of the enantiomers of *trans*-β-lactam ureas using acetonitrile as the mobile phase.

**Figure 7:**
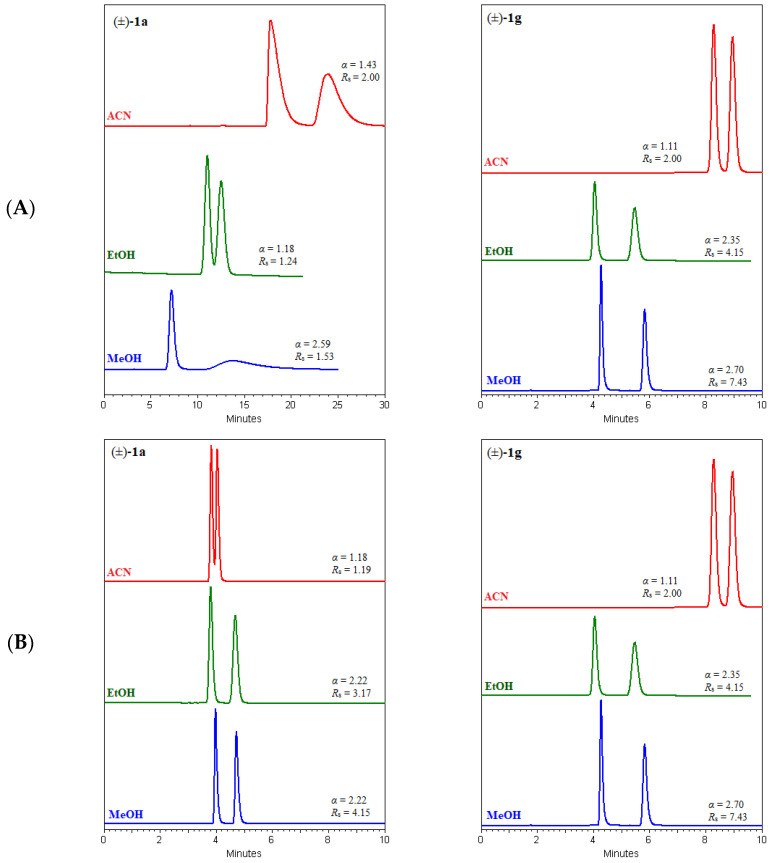

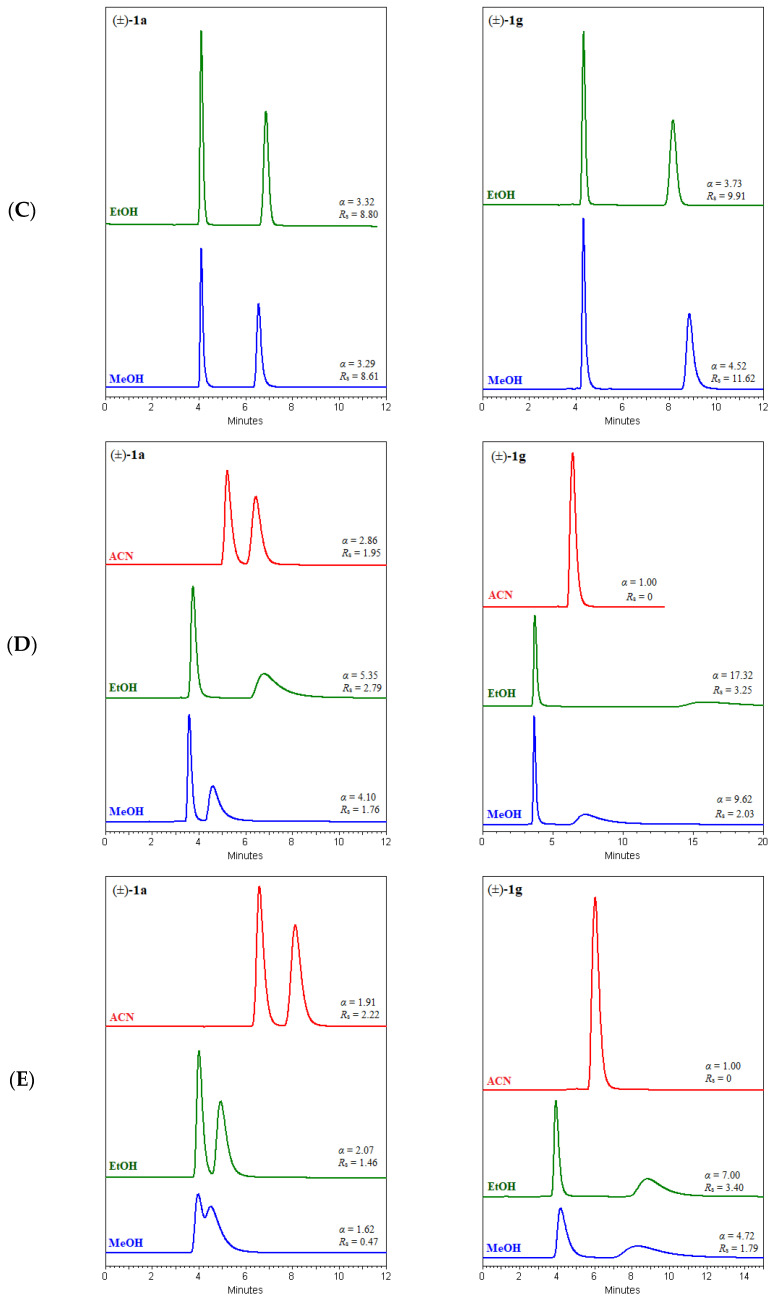

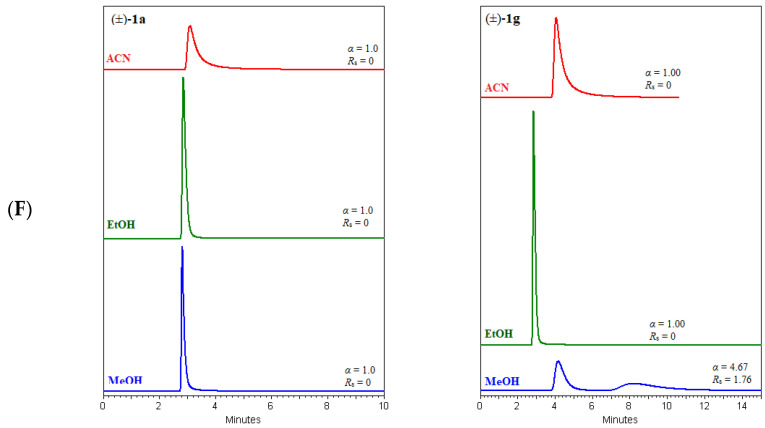
Chromatograms for the enantioselective separations of (±)-*trans*-β-lactam ureas **1a** and **1g** on Chiralpak AD-3 (**A**), Chiralcel OD-3 (**B**), Chirallica PST-10 (**C**), Chirallica PST-7 (**D**), Chirallica PST-8 (**E**), and Chirallica PST-9 (**F**) columns in the polar organic mode, MeOH (**―**), EtOH (**―**), and ACN (**―**).

Chromatograms of (±)-*trans*-β-lactam urea with phenyl group 1a and 3-chloro-4-methylphenyl group **1g** attached to the nitrogen atom of the ureido group are shown in Figure 7. The enantiomers of compound **1a** and **1g** were partially or baseline separated on the columns Chiralpak AD-3 column, Chiralcel OD-3, Chirallica PST-7, Chirallica PST-8, and Chirallica PST-10, with methanol and ethanol as the mobile phase. On the Chirallica PST-9 column, compound **1g** was separated only in methanol, while compound **1a** was not separated with either methanol or ethanol. In acetonitrile, compound **1a** was separated on the amylose-based Chiralpak AD-3 column and on its cellulose and levan analog, the Chiralcel OD-3 and Chirallica PST-7 columns, as well as on the Chirallica PST-8 column. In the same solvent, the enantiomers of compound **1g** were not separated on all three levan columns (Chirallica PST-7, Chirallica PST-8, and Chirallica PST-9), but very good enantioseparation was observed on the other columns tested (Chiralcel OD-3 and Chiralpak AD-3). The chiral recognition of these two β-lactam compounds (**1a** and **1g**) on the six polysaccharide CSPs tested depends on the differences in the supramolecular structure of these selectors, the type of substituent attached to the nitrogen atom of the ureido group, and the type of mobile phase used (methanol, ethanol, or acetonitrile).

In the showcase of enantioseparation of (±)-*trans*-β-lactam ureas **1a** and **1g**, the differences in the supramolecular structure of amylose, cellulose, and levan are clearly evident. Cellulose has a supramolecular structure without twists. The helical three-dimensional structure of amylose and levan favors stronger non-covalent interactions with the second eluting enantiomer. Also, as demonstrated in this work, the application of polar mobile phases on polysaccharide columns has an important influence on enantioseparation due to the formation of hydrogen bonds [63,64]. This is why the appearance of the peak of the second eluting enantiomer is broadened. 

## 3. Materials and Methods

### 3.1. Materials

The eight racemic β-lactam ureas were prepared in our laboratory by the addition of the corresponding isocyanate to (±)-*trans*-3-amino-β-lactam, which was prepared in three reaction steps [65].

Levan (Timothy grass, DP = 60) was purchased from Megazyme (Wicklow, Ireland). Dry pyridine, aroyl chlorides, aroyl isocynates, acetic acid, diethylamine, and Celitexxx were purchased from Sigma-Aldrich (Steinheim, Germany). Dichloromethane, methanol, 2-propanol, *n*-hexane, and acetonitrile were purchased from J.T. Baker (Dovenport, Holland) and distilled before use. Daisogel SP-1000-5-C1 was purchased from Daiso Co. Ltd. (Osaka, Japan). 

Gradient-grade methanol (MeOH) was purchased from Honeywell (Seelze, Germany).

The empty stainless-steel HPLC columns, dimensions 250 × 4.6 mm I.D., were purchased from Knauer Gmbh (Berlin, Germany). Woven wire mesh sieve (frame diameter 200 mm, mesh size 20 μm) and sieve accessories were purchase from Retsh GmbH (Haan, Germany).

Polysaccharide-type chiral columns with identical dimensions (250 mm × 4.6 mm I.D., 3 μm particle size), Chiralpak AD-3 (based on amylose *tris*(3,5-dimethylphenylcarbamate)) and Chiralcel OD-3 (based on cellulose *tris*(3,5-dimethylphenylcarbamate)), were purchased from Daicel Chiral Technologies Co., Ltd. (Tokyo, Japan). Chirallica PST-10 (based on cellulose *tris*(4-methylphenylcarbamate) was prepared at Ruđer Bošković Institute (Zagreb, Croatia).

### 3.2. Preparation of Cellulose tris(4-Methylcarbamate) CSP

Cellulose predried in an electric oven (1.0 g), dry pyridine (20 mL), and *p*-tolyl isocyanate (5 mL) were mixed together and heated at an oil-bath temperature of 90–100 °C for 48 h under an argon atmosphere. The reaction mixture was cooled to room temperature, the product was precipitated with methanol, filtered off, washed with methanol, and dried in an electric oven for 24 h. Product yield: 2.46 g of a white powdery substance.

### 3.3. Preparation of Levan Carbamate Derivatives

#### 3.3.1. Levan *tris*(3,5-Dimethylphenylcarbamate)

Levan predried in an electric oven (1.0 g), dry pyridine (20 mL), and 3,5-dimethylphenyl isocyanate (5 mL) were mixed together and heated at an oil-bath temperature of 90–100 °C for 48 h under an argon atmosphere. The reaction mixture was cooled to room temperature, the product was precipitated with methanol, filtered off, washed with methanol, and dried in an electric oven for 24 h. The crude product was mixed with dichloromethane (75 mL) and stirred on a magnetic stirrer for 60 min. The resulting suspension was separated by centrifuge, and the supernatant was evaporated to dryness. The dry residue was triturated with methanol and dried in an electric oven for 24 h. Product yield: 3.51 g of off-white powdery substance.

#### 3.3.2. Levan *tris*(4-Methylphenylcarbamate)

Levan predried in an electric oven (1.0 g), dry pyridine (20 mL), and *p*-tolyl isocyanate (5 mL) were mixed together and heated at an oil-bath temperature of 90–100 °C for 48 h under an argon atmosphere. The reaction mixture was cooled to room temperature, the product was precipitated with methanol, filtered off, washed with methanol, and dried in an electric oven for 24 h. Product yield: 2.46 g of a white powdery substance.

#### 3.3.3. Levan *tris*(1-Naphthylcarbamate)

Levan predried in an electric oven (1.0 g), dry pyridine (20 mL), and 1-naphthyl isocyanate (5.3 mL) were mixed together and heated at an oil-bath temperature of 90–100° C for 48 h under an argon atmosphere. The reaction mixture was cooled to room temperature, the product was precipitated with methanol, filtered off, washed with methanol, and dried in an electric oven for 24 h. Product yield: 3.23 g of a white powdery substance.

#### 3.3.4. MALDI-TOF MS Measurement

The MALDI-TOF MS experiments were performed on an Autoflex speed MALDI-TOF/TOF (Bruker Daltonics, Waldbronn, Germany) instrument operating in positive linear mode in the *m*/*z* range of 1000–12,000 Da. A solid-state laser (SmartBeam, medium, Waldbronn, Germany) was used. Samples of native and derivatized polysaccharide (1 mg mL^−1^ chloroform solution) were loaded with a fixed amount (0.5 μL) onto an anchor chip 386 MALDI plate by a “dried-droplet” method. Samples were then loaded with 0.5 µL of CHCA matrix (α-cyano-4-hydroxycinnamic acid, 5 mg mL^−1^ dissolved in 30% ACN, 70% H_2_O/0.1% TFA) and dried.

In general, spectra were obtained by accumulating 1000 laser shots for quantification. The delayed extraction time was set to 120 ns.

#### 3.3.5. SEM Characterization of Levan-Based CSPs

Scanning electron microscopy (SEM) of synthesized levan CSPs was carried out using a JOEL JSM-7000F field-emission scanning electron microscope.

### 3.4. Preparation of Levan Chiral Stationary Phases

The appropriate levan derivative was dissolved in dichloromethane. The solution was added to depolarized silica gel (3.00 g), and the mixture was sonicated for a few minutes. The solvent was removed with a rotary evaporator. The resulting chiral stationary phase was suspended in 2-propanol and passed through a sieve. The suspension was then filtered, and the separated chiral stationary phase was dried overnight in an electric dryer. After drying, the stationary phase was packed into the stainless-steel column (250 mm × 4.6 mm I.D.), using a conventional high-pressure slurry packing technique with a Knauer pneumatic HPLC pump (Knauer GmbH, Berlin, Germany).

### 3.5. HPLC Analysis

HPLC analyses were performed using an Agilent 1200 Series system (Agilent Technologies GmbH, Germany) consisting of a vacuum degasser, a quaternary pump, a thermostated column compartment, an autosampler, and a variable wavelength detector. The mobile phase was 100% MeOH. All experiments in the polar organic mode were performed under isocratic conditions at a flow rate of 1.0 mL min^−1^ and a column temperature of 30 °C. UV detection was performed at 254 nm. Agilent EZChrom Elite software version 3.1.7 (Agilent Technologies, Waldbronn, Germany) was used for the data analysis. The injection volume was 20 μL, and two parallel measurements were performed in all cases. The sample solutions of β-lactam ureas **1a**–**g** were prepared by dissolving in the appropriate amount of MeOH (*c* = 0.5 mg mL^−1^) and filtered through an RC-45/25 Chromafil^®^ Xtra 0.45 μm syringe filter (Macherey-Nagel GmbH & Co. KG, Düren, Germany).

The retention factors of the first and second eluted enantiomers (*k*_1_ and *k*_2_) were calculated according to the following equations:

*k*_1_ = (*t*_R1_ − *t*_0_)/*t*_0_, (1)

*k*_2_ = (*t*_R2_ − *t*_0_)/*t*_0_, (2)

where *t*_0_ is the retention times of the non-retained solute; and t_R1_ and t_R2_ are the retention times of the first and second eluted enantiomers, respectively. 

The enantioseparation factor (*α*) was calculated using the following equation:

*α* = *k*_2_/*k*_1_, (3)

where *k*_1_ and *k*_2_ are the retention factors of the first and second eluted enantiomers, respectively. By definition, the selectivity is always greater than one because, if α is equal to one, the two peaks are co-eluting (i.e., their retention factor values are identical) [65]. 

The resolution (*R*_s_) was calculated using the following equation:

*R*_s_ = 2(*t*_R2_ − *t*_R1_)/(*w*_b1_ + *w*_b2_), (4)

where *t*_R1_ and *t*_R2_ are the retention times of the first and second eluted enantiomers, respectively; and *w*_b1_ and *w*_b2_ are the baseline peak widths of the first and second eluted enantiomers, respectively. 

For the determination of dead time (*t*_0_), i.e., the retention time of a non-adsorbing component, 1,3,5-tri-*tert*-butylbenzene was used.

## 4. Conclusions

The enantioseparation of (±)-*trans*-β-lactam ureas **1a**–**h** was performed on amylose-, cellulose-, and new levan-based CSPs in the polar organic mode, using pure methanol, ethanol, and acetonitrile. The alcohol polar organic mode used on polysaccharide-based CSPs proved to be an excellent choice for the rapid and highly efficient chiral separation of the enantiomers of studied *trans*-β-lactam ureas **1a**–**h**. Of the six polysaccharide columns tested, Chiralcel OD-3, Chirallica PST-7, Chirallica PST-8, and Chirallica PST-10 showed great chiral recognition ability towards β-lactam compounds **1a**–**h** in methanol and ethanol. The Chiralpak AD-3 and the Chiralcel OD-3 columns appear to be the most suitable for the separation of the enantiomers of *trans*-β-lactam ureas using acetonitrile as the mobile phase.

Of the newly prepared levan-based CSPs, only the levan *tris*(1-naphthylcarbamate) CSP (Chirallica PST-9) did not show a satisfactory chiral recognition ability for the tested chiral *trans*-β-lactam ureas **1a**–**h**, as it was able to separate only four of them in methanol, and no separation was observed in ethanol and acetonitrile.

## Figures and Tables

**Table 1:** Chromatographic data: retention factor of the first eluted enantiomers (*k*_1_), selectivity factor (*α*), and resolution (*R*_s_) for (±)-*trans*-β-lactam ureas **1a**–**h** on the polysaccharide-type columns in polar organic mode.

		MeOH			EtOH			ACN		
β-Lactam Urea	Column	*k*_1_	*α*	*R*_s_	*k*_1_	*α*	*R*_s_	*k*_1_	*α*	*R*_s_
**1a**	Chiralpak AD-3	1.28	2.59	1.53	2.86	1.18	1.24	3.97	1.43	2.00
	Chiralcel OD-3	0.18	2.22	4.15	0.23	2.22	3.17	0.38	1.18	1.19
	Chirallica PST-10	0.35	3.29	8.61	0.41	3.32	8.80	-	-	-
	Chirallica PST-7	0.10	4.10	1.76	0.23	5.35	2.79	0.14	2.86	1.95
	Chirallica PST-8	0.29	1.62	0.47	0.28	2.07	1.46	0.35	1.91	2.22
	Chirallica PST-9	0.08	1.00	0	0.34	1.00	0	0.03	1.00	0
**1b**	Chiralpak AD-3	0.95	1.95	2.54	1.04	2.77	6.57	1.05	1.72	1.80
	Chiralcel OD-3	0.21	1.48	1.79	0.20	1.45	1.14	0.69	1.10	0.61
	Chirallica PST-10	0.27	2.63	5.88	0.28	2.36	4.62	-	-	-
	Chirallica PST-7	0.12	1.83	0.95	0.15	2.67	1.62	0.24	1.46	0.94
	Chirallica PST-8	0.23	1.57	0.45	0.17	1.88	1.02	0.18	1.58	0.99
	Chirallica PST-9	0.07	1.00	0	0.31	1.00	0	0.10	1.00	0
**1c**	Chiralpak AD-3	1.6	2.47	1.44	1.81	1.79	3.94	2.42	1.31	0.93
	Chiralcel OD-3	0.21	2.48	5.23	0.23	2.57	3.90	0.81	1.20	1.95
	Chirallica PST-10	0.45	3.51	9.65	0.42	4.24	10.89	-	-	-
	Chirallica PST-7	0.16	6.25	2.13	0.25	11.2	3.41	0.90	1.34	1.68
	Chirallica PST-8	0.43	1.02	0.93	0.35	2.91	2.17	0.38	1.61	1.71
	Chirallica PST-9	0.07	1.00	0	0.30	1.00	0	0.12	1.00	0
**1d**	Chiralpak AD-3	2.13	2.77	2.14	3.71	1.30	2.00	1.76	1.71	1.48
	Chiralcel OD-3	0.22	2.27	4.62	0.25	2.36	3.60	1.05	1.16	1.81
	Chirallica PST-10	0.39	3.56	9.33	0.43	3.42	9.08	-	-	-
	Chirallica PST-7	0.19	3.74	1.81	0.31	5.84	2.78	1.22	1.61	2.70
	Chirallica PST-8	0.45	1.51	0.20	0.39	1.87	1.34	0.47	2.09	2.73
	Chirallica PST-9	0.07	1.00	0	0.32	1.00	0	0.20	1.00	0
**1e**	Chiralpak AD-3	1.98	2.55	2.19	3.87	1.69	4.04	1.02	2.25	1.81
	Chiralcel OD-3	0.29	2.17	5.62	0.39	2.08	3.73	1.28	1.13	1.24
	Chirallica PST-10	0.53	3.08	9.19	0.68	2.63	8.47	-	-	-
	Chirallica PST-7	0.15	3.60	1.71	0.34	6.29	2.78	0.99	1.56	1.92
	Chirallica PST-8	0.45	1.76	0.66	0.40	2.03	1.58	0.44	1.89	1.83
	Chirallica PST-9	0.46	1.76	0.60	0.31	1.00	0	0.27	1.00	0
**1f**	Chiralpak AD-3	4.35	1.00	0	2.84	2.92	6.06	1.34	2.37	1.46
	Chiralcel OD-3	0.26	2.27	5.26	0.27	2.11	2.99	1.83	2.54	2.20
	Chirallica PST-10	0.40	3.33	8.54	0.44	2.84	6.95	-	-	-
	Chirallica PST-7	0.13	8.54	2.00	0.20	18.90	2.81	0.80	1.56	1.76
	Chirallica PST-8	0.37	3.16	1.27	0.27	3.96	2.41	0.24	1.00	0
	Chirallica PST-9	0.37	3.11	1.21	0.29	1.00	0	0.34	1.00	0
**1g**	Chiralpak AD-3	1.00	1.24	0.30	1.79	1.00	0	1.21	2.40	1.66
	Chiralcel OD-3	0.27	2.70	7.43	0.30	2.53	4.15	1.98	1.11	2.00
	Chirallica PST-10	0.42	4.52	11.62	0.48	3.73	9.91	-	-	-
	Chirallica PST-7	0.13	9.62	2.03	0.22	17.32	3.25	0.40	1.00	0
	Chirallica PST-8	0.36	4.72	1.79	0.26	7.00	3.40	0.24	1.00	0
	Chirallica PST-9	0.36	4.67	1.76	0.30	1.00	0	0.36	1.00	0
**1h**	Chiralpak AD-3	2.02	1.00	0	1.78	1.00	0	1.35	2.21	1.71
	Chiralcel OD-3	0.29	2.55	7.13	0.29	2.59	4.16	1.97	2.22	1.13
	Chirallica PST-10	0.50	4.04	11.32	0.50	3.62	9.88	-	-	-
	Chirallica PST-7	0.19	6.79	1.94	0.23	16.91	3.30	0.33	1.00	0
	Chirallica PST-8	0.58	2.57	1.25	0.26	7.04	3.39	0.28	1.00	0
	Chirallica PST-9	0.58	2.55	1.20	0.29	1.00	0	0.31	1.00	0

Flow rate, 1 mL min^−1^; detection at UV 254 nm; column temperature 30 °C. The Chirallica PST-10 column was not tested under ACN due to the high column back pressure.

## Data Availability

Data are contained within the article.
